# Protein arginine methyltransferase 5 (PRMT5) dysregulation in cancer

**DOI:** 10.18632/oncotarget.26404

**Published:** 2018-11-30

**Authors:** Harshita Shailesh, Zain Z. Zakaria, Robert Baiocchi, Saïd Sif

**Affiliations:** ^1^ Department of Biological and Environmental Sciences, College of Arts and Sciences, Qatar University, Doha, Qatar; ^2^ Division of Hematology, Department of Internal Medicine, The Ohio State University, Columbus, Ohio, USA

**Keywords:** PRMT5, histone arginine methylation, tumor suppressors, proliferative signaling, metabolic dysregulation

## Abstract

Protein arginine methyltransferases (PRMTs) are known for their ability to catalyze methylation of specific arginine residues in a wide variety of cellular proteins, which are involved in a plethora of processes including signal transduction, transcription, and more recently DNA recombination. All members of the PRMT family can be grouped into three main classes depending on the type of methylation they catalyze. Type I PRMTs induce monomethylation and asymmetric dimethylation, while type II PRMTs catalyze monomethylation and symmetric dimethylation of specific arginine residues. In contrast, type III PRMTs carry out only monomethylation of arginine residues. In this review, we will focus on PRMT5, a type II PRMT essential for viability and normal development, which has been shown to be overexpressed in a wide variety of cancer cell types, owing it to the crucial role it plays in controlling key growth regulatory pathways. Furthermore, the role of PRMT5 in regulating expression and stability of key transcription factors that control normal stem cell function as well as cancer stem cell renewal will be discussed. We will review recent work that shows that through its ability to methylate various cellular proteins, PRMT5 functions as a master epigenetic regulator essential for growth and development, and we will highlight studies that have examined its dysregulation and the effects of its inhibition on cancer cell growth.

## INTRODUCTION

Normal cell growth and development rely on the proper regulation and interplay of many pathways that control cell growth and division, as well as cell differentiation and death. Over the last two decades, it has become abundantly clear that modification of chromatin plays a central role in triggering the appropriate gene expression programs required during different stages of cell growth and development. Among the various modifications that affect chromatin is arginine methylation, which has been implicated in a growing list of cellular processes including signaling, transcription, RNA processing, DNA recombination and repair [1–6]. Protein arginine methyltransferases (PRMTs) catalyze methylation of specific arginine residues by transferring the methyl group from S-adenosylmethionine (SAM) to the guanidine nitrogen of arginine, resulting in either ω-N^G^-monomethylation or ω-N^G^, N^G^-asymmetric dimethylation and ω-N^G^, N'^G^-symmetric dimethylation. Based on the type of modification introduced, PRMTs can be classified into three groups: Type I (PRMT 1, 2, 3, 4, 6 and 8) catalyze monomethylation and asymmetric dimethylation, type II (PRMT5 and PRMT9) catalyze monomethylation and symmetric dimethylation, while type III (PRMT7) carry out monomethylation only [7–14]. It is worth noting that PRMT7 was initially classified as a type II PRMT, based on findings by Lee *et al.* (2005) [15]; however, a study by Zurita-Lopez *et al.* (2012) showed that PRMT7 exclusively catalyzes monomethylation of arginine residues in substrate proteins [16].

**Figure 1:**
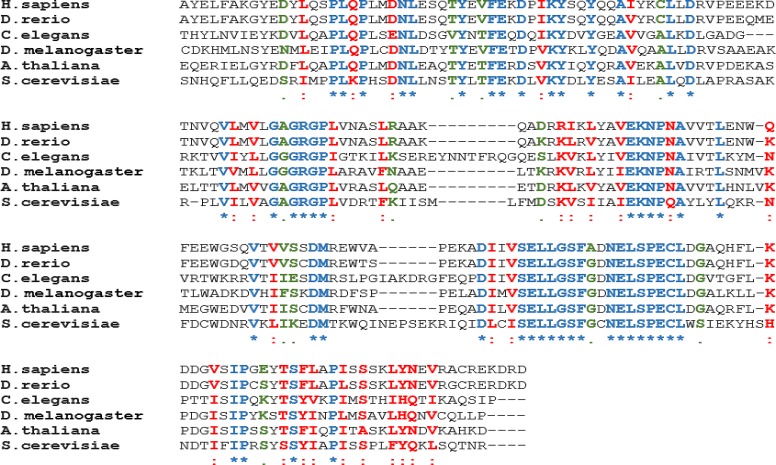
PRMT5 is highly conserved from yeast through human Amino acid sequences from the indicated species have been aligned using the Clustal Omega software. The conserved sequences are highlighted using different colors and symbols (Blue star represents conserved identical residues, red colon represents conserved highly similar residues; and black bullet represents moderately similar residues). Positions of amino acids used for *H. sapiens* (296–493), *D. rerio* (292–489), *C. elegans* (348–554), *D. melanogaster* (272–466), *A. thaliana* (302–496), and *S. cerevisiae* (317–518).

This review will focus on work that has been done recently to enhance our understanding of the biological functions of PRMT5. PRMT5 is ubiquitously expressed in all eukaryotes and its catalytic domain is highly conserved from yeast through humans (Figure 1). Based on its ability to bind the Janus kinase protein, PRMT5 was initially called Janus kinase binding protein [17]. However, later studies characterized its symmetric methylation activity [14], and showed that through its association with human SWI/SNF chromatin remodeling complexes, PRMT5 can symmetrically methylate histones H3 and H4 and regulate transcription of a specific set of target genes [18–20]. PRMT5-catalyzed histone H3 arginine 8 (R8) and H4R3 symmetric dimethylation has been shown to repress expression of several tumor suppressor genes such as *Suppressor of Tumorigenicity 7* (*ST7*), *retinoblastoma* (*RB*) family of tumor suppressors, and *Protein Tyrosine Phosphatase Receptor-type O* (*PTPROt*) [18, 21]. In a more recent study, PRMT5 has been shown to be overexpressed in a large cohort of human gastric tumors and to contribute to increased recruitment of DNA methyltransferase 3A (DNMT3A) to the promoter region of the tumor suppressor gene, *Iroquois homeobox 1 (IRX1)*, in gastric cancer cells. Furthermore, PRMT5 knock down in gastric cancer cell lines results in attenuated cell growth and reduced metastasis [22]. Regardless of whether the overexpressed PRMT5 interacts with a nucleosome remodeling complex or a DNA-modifying enzyme, its inhibition mitigates tumorigenesis and enhances cell death.

Although extensive evidence indicates that PRMT5-induced H3R8 and H4R3 methylation leads to transcriptional repression of its target genes, cellular differentiation studies have shown that PRMT5-induced histone methylation is also involved in transcriptional activation. For instance, in concert with cooperator of PRMT5 (COPR5), PRMT5 has been shown to be involved in the control of adipocyte progenitor cell fate through transcriptional repression of the Wingless/Integration 1 (Wnt) target gene, *Delta Like Non-Canonical Notch Ligand 1* (*Dlk-1*) [23]. However, in complex with the transcription factor Peroxisome Proliferative-Activated Receptor γ2 (PPARγ2), PRMT5 induces H3R8 methylation in the promoter region of adipogenic differentiation genes, and facilitates their transcription to promote adipogenesis [24], suggesting that PRMT5 may act either as a transcriptional activator or repressor.

Further evidence in support of the activator role played by PRMT5 comes from studies of the *Fibroblast-derived Growth Factor Receptor-3* (*FGFR-3*) and *eukaryotic elongation Initiation Factor-4E* (*eIF4E*) genes in colorectal cancer [25]. In this study, the authors measured PRMT5 levels in both patient-derived cell lines as well as primary tumors, and investigated the impact of PRMT5-mediated histone H3 and H4 methylation on *FGFR-3* and *eIF4E* expression. The results from these experiments revealed that PRMT5-induced H3R8 and H4R3 symmetric methylation increases transcription of *FGFR-3* and *eIF4E*, and that PRMT5 knockdown reduced the levels of methylated H3R8 and H4R3 as well as expression of both genes. More recently, Deng *et al.* (2017) showed that PRMT5 stimulates expression of the androgen receptor (AR) in prostate cancer cells. In this study, co-immunoprecipitation results showed that PRMT5 interacts with both BRG1 and Sp1 to induce H4R3 symmetric methylation in the promoter region of the *androgen receptor* gene, resulting in its activation and enhanced cell growth. The authors also showed that inducible knockdown of PRMT5 inhibits growth of AR-positive but not AR-negative prostate cancer cells as well as xenograft tumors in an AR-dependent manner. Similarly, pharmacological inhibition of PRMT5 decreased symmetric methylation of H4R3, expression of AR in AR-positive prostate cancer cells, and reduced cancer cell growth [26].

**Figure 2:**
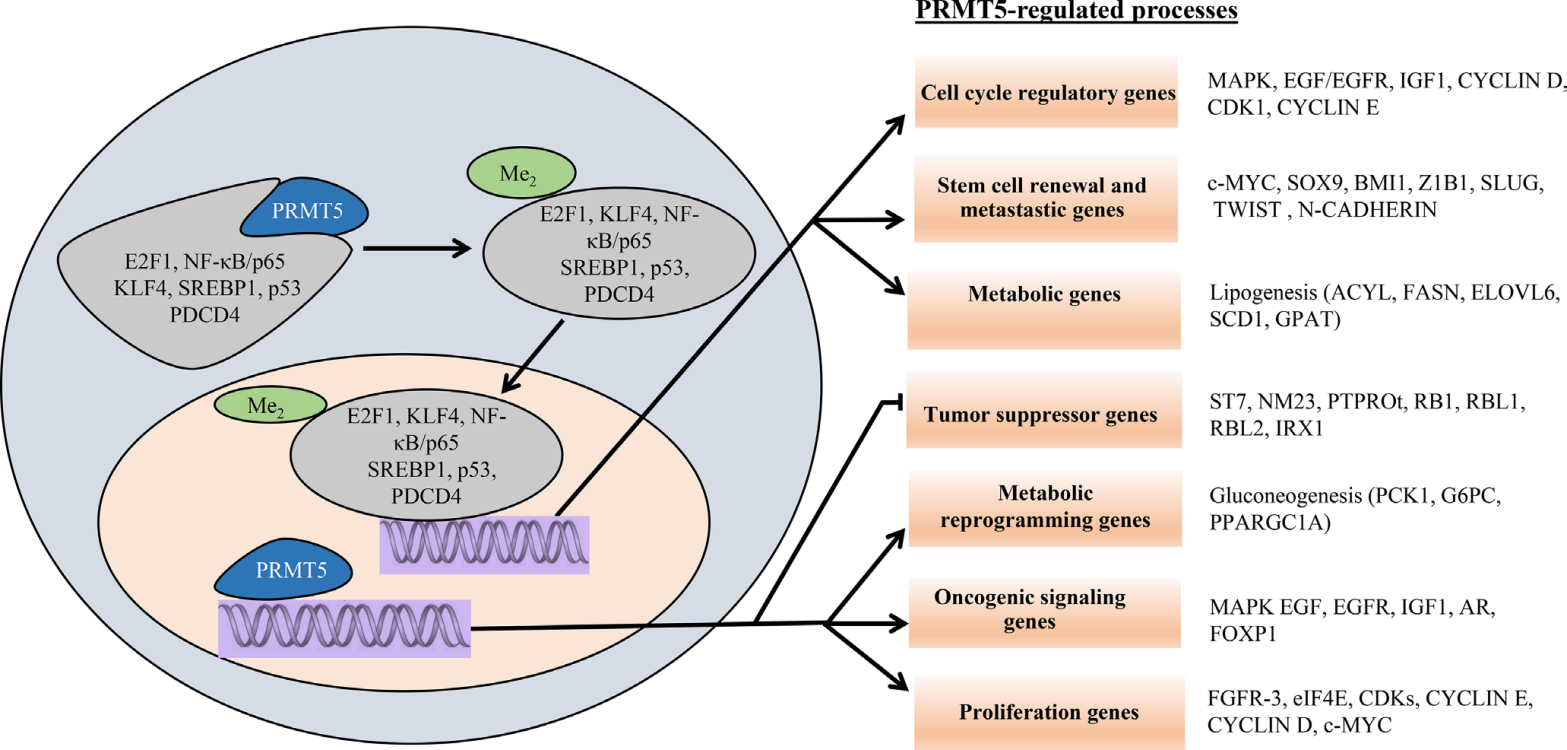
Schematic model of PRMT5-regulated cellular processes Cytosolic PRMT5 induces arginine methylation of various transcription factors, which translocate into the nucleus and regulate expression of their respective target genes. Nuclear PRMT5 is also directly recruited to the promoter regions of specific target genes to enhance cellular proliferation and oncogenesis.

Besides its ability to methylate histones, PRMT5 is also capable of methylating several important transcription factors (Table 1), highlighting its central role in cellular regulation. PRMT5 can methylate p53 and modify its DNA binding activity, which in turn triggers a change in the p53-controlled gene expression program [27]. PRMT5 has also been shown to methylate N-MYC and alter its protein stability as well as enhance its oncogenic activity in neuroblastoma [28]. Two other important transcription factors modified by PRMT5 include E2F-1 and NF-κB/p65, which once methylated, become more efficient at inducing target gene expression [29, 30]. The spectrum of PRMT5 target proteins is not limited to nuclear transcription factors, but also includes cytoplasmic proteins such as golgin, ribosomal protein S10 (RPS10), and rapidly accelerated fibrosarcoma (RAF) protein kinase [31–33]. Thus, in addition to its ability to directly regulate its own target genes, PRMT5 is able to influence global gene expression indirectly through symmetric methylation of key transcription factors, thereby impacting cell growth, proliferation, and differentiation (Figure 2).

**Table 1:** PRMT5 methylated proteins

PRMT5 substrates	Biological function	Citations
Histones (H3R2, H3R8, H4R3), SPT5, FCP1, MBD2, KAP1, N-MYC PAX3, RNA polymerase II	Regulation of transcription	[18, 28, 55, 74–79]
SmD3, CF 1(m)68	Post transcriptional regulation	[80, 81]
FGF-2, RPS10, G3BP1	Regulation of Translation	[32, 82, 83]
p53, ASK1, PDCD4, CRN5	Regulation of apoptosis	[6, 27, 50, 84]
EGFR, RAF, E2F-1, FEN1, Androgen receptor, srGAP2, PDGFRα	Regulation of cell proliferation, migration, differentiation, and survival	[33, 46, 64, 85–87]
NF-κB/p65, HOXA9	Immune response regulation	[71, 73]
MBP, GM130	Cellular integrity	[14, 31]
Piwi proteins, Rad9	Genome stability	[88, 89]
SHP, TDH, SREBP1	Metabolic regulation	[51, 90, 91]

### PRMT5 and control of proliferative signaling in cancer

**Figure 3:**
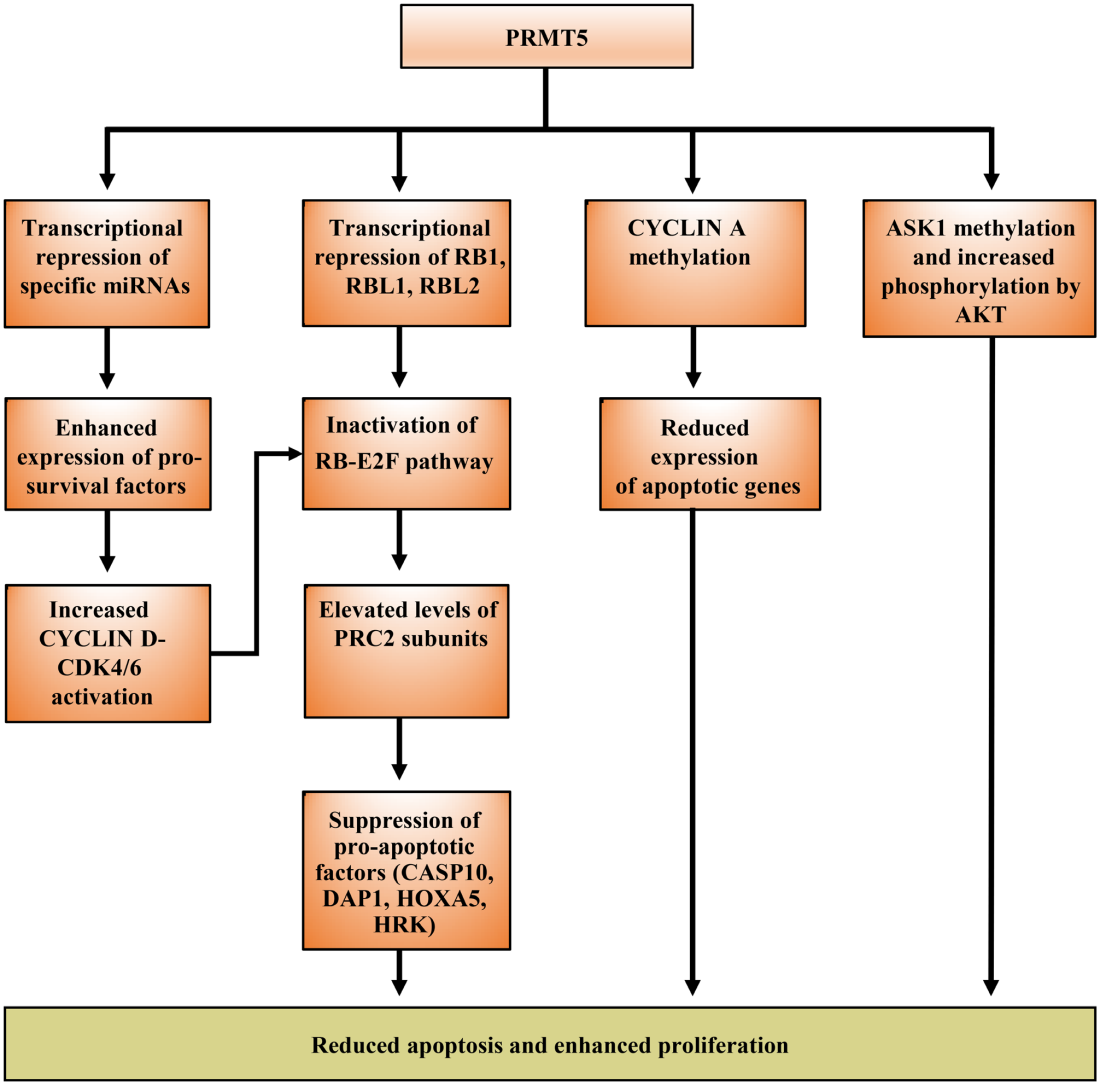
Elevated expression of PRMT5 results in reduced cell death and enhanced proliferation PRMT5 regulates apoptosis via different mechanisms including transcriptional repression to alter expression of specific micro-RNAs and the RB family of tumor suppressor genes, as well as post-translational modification of key regulatory factors such as CYCLIN A and ASK1 to reduce apoptosis.

A large number of studies have documented PRMT5 overexpression in cancers of different types and aggressiveness including B and T cell lymphoma [19, 20, 34], metastatic melanoma [35], neuroblastoma and glioblastoma [28, 36, 37], germ cell tumors [38], ovarian cancer [39], nasopharyngeal cancer [40], breast cancer [41], colorectal cancer [25], and gastric cancer [42]. However, details of the mechanisms by which PRMT5 levels are altered were unknown until a study by Pal *et al.* (2007) showed that down regulation of microRNAs (miR) 92b and 96 was responsible for enhanced translation of PRMT5 mRNA in various B cell lymphoma types [19]. In another study by Alinari *et al.* (2015), the authors showed that, through a negative feedback loop and in combination with NF-κB/p65 and HDAC3, PRMT5 binds to the *miR96* promoter and suppresses its transcription (Figure 3) [21]. These findings argue that enhanced PRMT5 expression is a direct result of posttranscriptional dysregulation, and that PRMT5 promotes its own expression through direct inhibition of a specific miRNA program.

Several reports have tied PRMT5 overexpression with cellular transformation [21, 34, 43]. While early studies showed that episomal expression of PRMT5 induces transformation of immortalized NIH3T3 fibroblasts *in vitro*, more recent work showed that PRMT5 inhibition in cancer cells results in growth arrest and death both *in vitro* and *in vivo* [44]. Evidence in support of the role played by PRMT5 in cellular transformation comes from the experiments carried out by Alinari and colleagues, in which the authors showed that upon EBV infection of normal B lymphocytes, PRMT5 protein levels increase as early as 4 to 8 days post-infection. What is more interesting, is that immediately after PRMT5 induction, the miR96 levels decreased and the levels of PRMT5 direct target genes such as *ST7* and *PTPROt* dropped significantly [21]. These results confirmed the relevance of the negative feed-back loop that exists between PRMT5 and miR96. Furthermore, when PRMT5 was inhibited with either a specific small molecule inhibitor, or silenced using short hairpin RNA-mediated knock down, the miR96 levels were restored and so was the level of its target genes. These findings indicate that PRMT5 plays an essential role in the control of cell growth and proliferation, and that its overexpression promotes cellular transformation.

Studies over the last decade have clearly shown that enhanced PRMT5 expression in cancer cells is linked with transcriptional silencing of its target tumor suppressor genes [18, 21]. One of the main growth regulatory pathways controlled by PRMT5 is the highly conserved RB-E2F pathway. PRMT5 regulates this pathway through direct methylation of histones H3R8 and H4R3 in the promoter region of *RB1*, *RBL1* and *RBL2* tumor suppressor genes (Figure 2). The net outcome of these modifications is transcriptional repression of all three tumor suppressor genes; however, only *RBL2* appears to be suppressed at the protein level [20]. Even though *RB1* and *RBL1* are not suppressed translationally, they are both inactivated via hyperphosphorylation by the CYCLIN D-CDK4/6 kinase complex (Figure 3) [34]. Here again, PRMT5 appears to contribute to CYCLIN D-CDK4/6 activation through suppression of miR96, which under normal conditions acts by suppressing CYCLIN D expression (Karkhanis and Sif, unpublished).

Among the many RB-E2F targets are genes that code for components of the Polycomb Repressor Complex (PRC) 2, which include Enhancer of Zeste Homolog (EZH) 2, Suppressor of Zeste (SUZ) 12 homolog, and Embryonic Ectoderm Development (EED). Previous work had shown that subunits of the PRC2 complex are transcriptionally regulated by the RB/E2F complex [45]. More recently, PRMT5 was shown to directly control transcriptional activity of members of the E2F family, such as E2F1, via methylation of specific arginine residues (Figure 2) [46], as well as indirectly by favoring hyperphosphorylation and inactivation of the RB family of tumor suppressor proteins (Figure 3) [34]. Therefore, a consequence of PRMT5-mediated inhibition of the RB family of tumor suppressor proteins is elevated expression of PRC2 subunits as well as increased PRC2 methyltransferase activity, which in turn promotes cancer cell growth and proliferation through suppression of anti-cancer genes including pro-apoptotic target genes *CASP10*, *DAP1*, *HOXA5*, and *HRK* [34]. In light of these results, PRMT5 is able to promote cancer cell growth by causing global chromatin changes through methylation of promoter histones H3R8 and H4R3, as well as by modifying specific arginine residues of key transcription factors including E2F1 and NF-kB/p65 [27].

A fascinating aspect of PRMT-induced arginine methylation is that different members of the PRMT family endow their target proteins with distinct properties. A good example of this regulation is illustrated by the mutually exclusive methylation of E2F1 by PRMT1 and PRMT5. In an elegant investigation led by La Thangue and co-workers, it was shown that PRMT1-mediated asymmetric dimethylation of E2F1 at arginine 109 (R109) enhances its transcriptional activity, which results in increased expression of E2F1 pro-apoptotic target genes during DNA damage [46]. In stark contrast, binding of CYCLIN A to E2F1 precludes PRMT1 binding, and promotes PRMT5-induced symmetric dimethylation of E2F1 at R111 and R113. As a result, symmetrically methylated E2F1 becomes bound by the Tudor domain protein p100-TSN, which suppresses its apoptotic activity and enhances its growth stimulating functions [46]. Thus, depending on the type of arginine modification introduced into E2F1, different growth outcomes ensue, further confirming the role played by PRMT5 in preventing apoptosis and promoting cell proliferation.

Another example of the relevant role played by PRMT5-mediatd arginine methylation in the control of genome stability and tumorigenesis is illustrated by the Kruppel-like factor 4 (KLF4), which is involved in regulating cell-fate decision and cell cycle progression [41]. In this instance, PRMT5 was shown to methylate KLF4 at R374, R376, and R377, which in turn inhibits KLF4 ubiquitylation by the VHL/VBC E3 ligase and increases its stability. KLF4 is a short-lived transcription factor involved in many cellular processes that control proliferation, genome stability, apoptosis, and stem cell renewal. Therefore, altering its stability will indirectly affect its several downstream targets. PRMT5 knock down or inhibition in different cancer cell lines results in increased KLF4 ubiquitylation and turnover, highlighting the crucial role played by PRMT5 in KLF4 protein stability. Furthermore, ectopic expression of PRMT5 or KLF4 in MCF-7 cells leads to cellular accumulation of methylated KLF4 protein, which reduces transcription of the pro-apoptotic gene *BAX*, thereby promoting cancer cell growth and survival. In accord with these results, breast cancer gene array profiling indicated that elevated expression of KLF4 in both MCF10A and MCF7 breast cancer cell lines upregulates transcription of several oncogenes and cell cycle activators such as MAPK, EGF/EGFR, IGF1, CYCLIN D2, CDK1, and CYCLIN E1. Other factors involved in regulating stem cell renewal and metastasis were also upregulated in KLF4 overexpressing breast cancer cells including c-MYC, SOX9, BMI1, Z1B1, SLUG, TWIST and N-CADHERIN. More importantly, analysis of clinical samples showed that the cellular levels of both PRMT5 and KLF4 were elevated in highly aggressive breast tumor tissues compared to adjacent normal tissues. These results clearly demonstrate that PRMT5 regulates key stem cell transcription factors through cross-talk between arginine methylation and ubiquitylation, and that it is a master epigenetic regulator capable of targeting multiple pathways to promote cancer cell growth and survival [41].

In line with its influence on proliferation and self-renewal of embryonic and adult stem cells, PRMT5 has recently been implicated in the regulation of breast cancer stem cell (BCSC) function [47]. The findings in this investigation showed that PRMT5 expression is elevated in BCSCs, and that its knock down reduces proliferation and self-renewal of BCSCs both *in vitro* and *in vivo*. Furthermore, histopathological analysis of excised tumors from NSG (NOD/Scid/IL-2Rγnull) mice injected with either control or PRMT5 knock down BCSCs showed that reduced PRMT5 expression results in a more differentiated and less aggressive breast cancer phenotype compared to control tumors, indicating that PRMT5 promotes breast cancer cell stemness by suppressing differentiation. While these initial experiments strongly suggested that PRMT5 is vital for growth and proliferation of BCSC, global RNA-Seq profiling in control and PRMT5 knock down BCSCs revealed differential expression of the *forkhead box protein 1* (*FOXP1*) transcription factor, which is known to be associated with normal and cancer stem cell function. Further characterization of the relationship between PRMT5 and *FOXP1* demonstrated that PRMT5 upregulates *FOXP1* expression through symmetric methylation of H3R2, which serves as a binding site for the WDR5 subunit of the SET1/MLL methyltransferase complex. Consistent with these recruitment studies, either inactivation of PRMT5 with the GSK591 inhibitor or disruption of the interaction between WDR5 and the SET/MLL1 complex triggered a dramatic decrease in H3K4 methylation and FOXP1 expression. These results, in combination with the inhibitory effects that FOXP1 knock down has on breast cancer cell growth both *in vitro* and in xenografts, imply that some of the tumor promoting properties of PRMT5 are mediated through FOXP1, and further suggest that PRMT5 inhibition might be a viable therapeutic option that should be explored in breast cancer treatment.

The role-played by PRMT5 in the onset and progression of cancer is becoming more and more clear as previous work by Wei and colleagues showed that ectopic expression of PRMT5 is associated with upregulation of the G1 phase cyclins D1, D2, and E1, as well as cyclin-dependent kinases (CDK) 4 and 6 [44]. Experiments in this study also showed that PRMT5 promotes cellular transformation by activating phosphatidylinositol 3-kinase (PI3K) and AKT (protein kinase B), which are known for their ability to suppress expression of pro-apoptotic genes and promote cell survival. A more recent study further confirms the pro-survival activity of PRMT5, which can methylate the apoptosis signal-regulating kinase 1 (ASK1) at R89 and promote its association with AKT [6]. Interaction of symmetrically methylated ASK1 with AKT results in its further phosphorylation at serine 83, which in turn attenuates its activity and inhibits apoptosis. Other evidence in support of the growth promoting activity of PRMT5 has been provided by experiments of Lim and co-workers, who showed that PRMT5 controls proliferation and survival through enhanced translation of oncogenes such as *c-MYC* and *CYCLIN D1* [48]. The mechanism by which PRMT5 operates in this case involves recruitment of eIF4E to the 5′-cap-binding region of the c-MYC and CYCLIN D1 mRNA. In a previous study, the relationship between PRMT5 and eIF4E was investigated, and the results suggested that PRMT5 enhances translation through regulation of eIF4E expression [25]. Taken together, these results indicate that PRMT5 promotes cellular proliferation through transcriptional, translational, and posttranslational control of key cell cycle regulators.

Although the list of PRMT5 associated proteins has grown significantly over the last few years, insight into the biological relevance of these interactions has only begun. Programmed Cell Death 4 (PDCD4) is a tumor suppressor protein whose expression has been shown to correlate with better survival in various cancers; however, upon interaction with PRMT5, PDCD4 becomes methylated at R110 and loses its tumor suppressor activity in MCF-7 cells [49]. Experiments that solidify the importance of interaction between PRMT5 and PDCD4 revealed that when either protein is mutated, the tumor promoting activity of the pair is lost, indicating that PRMT5-mediated PDCD4 methylation is crucial for enhanced tumor growth. Moreover, patients overexpressing both PRMT5 and PDCD4 show poor survival rate in comparison to patients expressing high PDCD4 levels and low levels of PRMT5 [49]. Increased PRMT5 levels have been found to correlate with poor prognosis in various cancer, and in a follow up study, the same group further validated the association of PRMT5 with PDCD4 [50]. In this latter study, it was shown that PDCD4 methylation promotes tumor cell viability during nutrient deprivation, and that this process is reversed upon recovery from starvation through phosphorylation of PDCD4 and it translocation to the nucleus. Collectively, these studies indicate that PRMT5 overexpression may lead to its interaction with both growth promoting and tumor suppressor proteins, thereby influencing their biological activities in such a way to favor cancer cell growth, survival and invasiveness.

### PRMT5 and metabolic dysregulation in cancer

Cancer cells alter their metabolic pathways in order to support rapid growth and proliferation, and most of the acquired changes are optimized to promote transformed cell growth. Besides altering pro-survival and cell death pathways, PRMT5 has been implicated in reprogramming of lipogenesis in cancer cells, and in modulation of the G1 to S phase checkpoint under high glucose concentration. The gene expression program responsible for biosynthesis of fatty acids, triglycerides, and phospholipids is tightly controlled by the transcription factors, sterol regulatory element-binding protein (SREBP) 1a and c [51]. Both transcription factors have been shown to interact with PRMT5 in HEK293 and HepG2 cell lines, highlighting the relevance of this association in increased lipogenesis of cancer cells. Furthermore, dependence on the PRMT5 catalytic activity for this interaction revealed in fact that PRMT5 symmetrically methylates SREBP1a on R321, which prevents its phosphorylation on serine 430 by GSK3β and increases its transcriptional activity, thereby enhancing lipogenesis in hepatocellular carcinoma [51]. PRMT5-mediated methylation not only prevents SREBP1a phosphorylation, but also inhibits its ubiquitination and degradation by the proteasome pathway. This was substantiated by mutational analysis, which showed that SREBP1a mutant R321K exhibits strong association with the ubiquitin ligase F-box and WD repeat domain-containing 7 (Fbw7), an event essential for SREBP1a degradation, compared to the SREBP1a double mutation R321K/S430A that blocked this interaction and stabilized SREBP1a.

Similarly, xenograft studies revealed that HepG2 cells expressing wild type SREBP1a induced tumors that were larger and heavier compared to cells expressing mutant R321K, which were less proliferative as measured by Ki67 expression. In addition, analysis of patient-derived hepatocellular carcinoma (HCC) samples revealed a strong correlation between the amount of SREBP1a symmetric methylation and aggressiveness of tumors as measured by size, stage and histologic grade. In another study, it was shown that SREBP1c overexpression correlates with high expression of lipogenic genes in clear cell Renal Cell Carcinoma (ccRCC), and that it is downregulated by the ring finger protein 20 (RNF20), which contains a ring finger-containing E3 ubiquitin ligase activity that promotes SREBP1c polyubiquitination and subsequent degradation [52]. Since SREBP1c has been shown to interact with PRMT5 in both HEK293 and HepG2, it is tempting to speculate that SREBP1c might also undergo the same type of symmetric methylation, which might enhance its stability and contribute to elevated lipid metabolism and cancer cell growth. These findings clearly show the important role played by PRMT5 in increasing lipogenesis of cancer cells by stabilizing SREBP1a, and perhaps also SREBP1c, and demonstrate a strong correlation between increased PRMT5-mediated SREBP protein methylation and aggressiveness of HCC.

Among the many changes cancer cells acquire as they become transformed is to use aerobic glycolysis in order to generate ATP instead of oxidative phosphorylation [53]. Cancer cells achieve this switch through upregulation of the ATPase Inhibitory Factor 1 (IF1), which can directly inhibit the H+-ATPase synthase, and down-regulation of the catalytic subunit of the H+-ATPase synthase (β-F1-ATPase). Addiction of cancer cells to glucose is accompanied by molecular changes that promote cancer cell growth. A recent study has shown that in the presence of high glucose, PRMT5 interacts with the cyclin dependent kinase 4 (CDK4) and promotes its release from the CDK inhibitor CDKN2A (p16^INK4a^), thereby facilitating G1-S transition in HCC cells [54]. PRMT5 has also been linked to metabolic reprogramming of gluconeogenic gene expression [55]. Under fasting conditions, hepatic glucose production is stimulated as a direct result of post-translational modification of two key factors, c-AMP response element binding (CREB) protein and CREB regulated transcriptional coactivator 2 (CRTC2), which participate in proper regulation of gluconeogenic target genes [56]. When blood glucose levels decrease, glucagon is released into the bloodstream by pancreatic alpha cells so that it induces activation of the cAMP-dependent protein kinase A (PKA), which in turn leads to direct CREB phosphorylation and indirect CRTC2 dephosphorylation. The ultimate result of this activation is association of CRTC2 with PRMT5, which symmetrically methylates histone H3R2 in the promoter region of gluconeogenic genes and promotes PKA-mediated CREB phosphorylation as well as target gene expression [55]. Therefore, it seems that PRMT5, through its ability to methylate either histone or non-histone proteins, is able to regulate metabolic pathways that are crucial for cell survival and proliferation.

Deletion of metabolic enzyme 5-methylthioadenosine phosphorylase (MTAP) is a frequently observed phenomenon in many cancers harboring *CDKN2A* (p16^INK4a^ and p14^ARF^) tumor suppressor gene deletion. The consequence of this frequent deletion is accumulation of methylthioadenosine (MTA), which selectively inhibits PRMT5 methyltransferase activity and sensitizes it to further inhibition [57]. What is interesting about the shRNA screen conducted in this study using 390 cancer cell lines, is that most of the PRMT5 interacting regulatory subunits, including methylosome protein 50 (MEP50/WDR77), methylosome subunit pICIn, and RIO kinase (RIOK1) were also found to be required for growth of MTAP-deficient cancer cells. These findings suggest that all cellular pools of PRMT5 are targeted for MTA-mediated inhibition, and that this metabolic vulnerability should be further exploited in MTAP-deleted cancer cells [57, 58].

### PRMT5 is required during development

Studies investigating the role played by PRMT5 during mouse embryonic development have shown that abundant levels of PRMT5 protein are found in the cytoplasm of mouse embryonic stem (ES) cells, where it interacts with STAT3 to maintain embryonic stem cell pluripotency by repressing transcription of differentiation genes such as *FGF5*, *GATA6*, *LHX1*, *FOX2*, *HOXA3*, *HOXA7* and *HOXD9* [59]. Furthermore, knockdown of PRMT5 in embryonic stem cells results in downregulation of key pluripotency genes such as *OCT4*, *NANOG* and *REX1*, whereas PRMT5 knockout in mouse models leads to abnormal embryonic growth followed by lethality [59]. These findings indicate that PRMT5 is an indispensable histone-modifying enzyme during embryonic development. More evidence in support of this notion comes from a study that shows that PRMT5 in complex with the Positive Regulatory Domain (PRDM) family transcription factor, Schwann Cell Factor 1 (SC1), plays a key role in maintaining the “stem-like” cellular state of neural stem cells of mouse cerebral cortex. In this instance, PRMT5 associates with SC1 to control neuronal stem cell differentiation by reducing expression of pro-mitotic genes such as *CYCLIN B* and *BUB1b* [60]. PRMT5 is required for neural stem cell survival, and its depletion in the central nervous system (CNS) of a *Nestin-Cre* transgenic mouse results in CNS developmental defects and lethality within 14 days after birth [61]. PRMT5 levels also increase gradually during postnatal brain growth, coinciding with the period of active myelination, highlighting its role in brain development [62]. The authors in this study also showed that PRMT5 is required for maintaining the methylation status of CpG islands in the promoter region of *Id2* and *Id4* leading to their silencing during glial cell differentiation.

Recent work by Scaglione *et al.* (2018) showed the functional relevance of PRMT5-mediated histone H4R3 symmetric methylation in oligodendrocyte differentiation and developmental myelination. Evaluation of PRMT5 mRNA demonstrated that its levels are high in proliferating, but not in differentiating, oligodendrocyte progenitor cells (OPCs). In addition, symmetric methylation of histone H4R3 was found to be enriched in the cytoplasm of proliferating OPCs and in the nucleus of differentiating OPCs. Interestingly, conditional deletion of PRMT5 in OPCs results in a severe hypomyelination at postnatal day 14 due to a decrease in the number of oligodendrocytes, but without any effect on proliferation of OPCs. These findings were further confirmed using either the PRMT5 inhibitor GSK591 or a PRMT5-specific CRISPR/Cas9 lentivirus, suggesting that PRMT5 is more important for survival and differentiation of OPCs than for their proliferation. Mechanistically, the authors determined, using both brain sections of PRMT5 mutant mice and *ex-vivo* primary cultures of OPCs, that PRMT5 regulates differentiation of OPCs through induced symmetric methylation of H4R3, which precludes acetylation of nearby lysine residues [63].

Consistent with these findings, Calabretta *et al.* (2018) showed in a different study that loss of PRMT5 (Oligo2-Cre) triggers early post-natal mortality and altered brain development, which is marked by hypomyelination and lack of mature oligodendrocytes (OL). The root cause of this deficiency stems from the fact that conditional PRMT5 inactivation in *PRMT5^FL/FL;Olig2Cre^* mice leads to decreased expression and plasma membrane localization of PDGFRα protein in the OL lineage. Similar results were observed in rat primary OPCs when PRMT5 was either knocked down, using a PRMT5-specific siRNA, or inhibited, using the PRMT5 specific inhibitor EPZ015666. Moreover, functional characterization revealed that PRMT5 interacts with and methylates PDGFRα on R554 within a Cbl E3 ligase binding site (aa 553-568). Thus, PRMT5-mediated PDGFRα methylation prohibits its degradation, and promotes self-renewal and proliferation of OPCs. However, as PRMT5 expression diminishes during OL differentiation, symmetric methylation of PDGFRα/R554 decreases, unmasking the Cbl E3 ligase binding site and triggering PDGFRα proteasome degradation [64].

More *in vivo* studies show that PRMT5 levels are elevated in mouse germ cells soon after sex determination during embryonic development, and its loss leads to cell death resulting in inefficient germ line development [65]. The role of PRMT5 in this case is to promote expression of meiosis-associated genes such as *STRA8*, *SCP3*, and *γH2AX* in mouse ovaries soon after sex determination, and downregulation of these genes after PRMT5 inactivation leads to inactivation of meiosis, thereby, resulting in germ cell loss during oogenesis. Similarly, conditional PRMT5 knock down in testes of PRMT5^Δ/f^;STRA8-Cre transgenic mice results in aberrant spermatogenesis, which is accompanied by reduced expression of meiotic genes such as *STRA8*, *SPO11*, *RAD51*, *SEP1*, *SEP3*, *DME1*, and *REC8*. This ultimately leads to meiotic arrest and death of germ cells in adult testes [65]. Unlike in embryonic stem cells, loss of PRMT5 does not reduce expression of pluripotent genes *OCT4*, *SOX2* and *NANOG* in germ cells. Instead, expression of these genes becomes elevated, indicating that PRMT5 plays cell specific roles during embryonic development [66]. In stark contrast, the role of PRMT5 in human embryonic life is not extensively studied. However, an investigation by Gkountela *et al.* (2014) showed that loss of PRMT5 in human embryonic stem cells does not affect transcriptional activity of pluripotency genes *OCT4*, *SOX2*, and *NANOG*, suggesting that PRMT5 is not involved in maintaining pluripotency of human stem cells. The same study also showed that PRMT5 plays a crucial role in promoting human embryonic stem cell proliferation via transcriptional repression of the p57^KIP2^ cell cycle inhibitor during self-renewing conditions [67]. Taken together, these studies indicate that PRMT5 serves as a key regulator of cellular reprogramming in murine embryonic stage, and that it possesses species- and cell-specific properties to regulate embryonic cell growth and development.

The role of PRMT5 extends beyond the germ cells, embryonic stem and neural stem cells compartments. PRMT5 plays an essential role in blood progenitor cell specification as demonstrated by the anemia and pancytopenia developed by conditional ablation of PRMT5 (*Mx1-Cre*) in adult hematopoietic stem cells (HSC) [68]. Lack of PRMT5 expression disrupts cell cycle progression in progenitor cells, leading to reduced expression and signaling of cytokine receptors in HSC. This in turn, results in increased p53 signaling and enhanced expression of its downstream targets [68]. Consequently, hematopoietic progenitor stem cells are lost and development of both B and T cell lineages are impaired [69], suggesting therefore, that PRMT5 is required for HSC maintenance and expansion. The impact of PRMT5 on the immune system has not been studied extensively; however, few studies have highlighted its involvement in modulating expression of immune response genes. PRMT5 along with its binding partner MEP-50 can associate with cyclin dependent kinases, CDK8 and CDK19, and methylate histone H4 in the promoter region of *IL-2* and *TNF-α*, which become transcriptionally repressed under normal conditions [70]. Thus, PRMT5 appears to be involved in regulating the acute phase response of innate and adaptive immunity through transcriptional control of *IL-2* and *TNF-α*.

PRMT5 has also been shown to promote transcription of pro-inflammatory genes in activated endothelial cells during inflammation by methylating several transcription factors. PRMT5-mediated methylation of Homeobox transcription factor 9 (HOXA9) in TNF-α activated endothelial cells promotes transcription of pro-inflammatory genes such as the cytokine-induced expression of E-selectin and Vascular Cell Adhesion Molecule 1 (VCAM-1), which help leukocyte adhesion and infiltration at the site of inflammatory lesions [71]. In a different study, Harris *et al.* (2014) showed that PRMT5 symmetrically methylates R30 and R35 of NF-κB/p65 in TNF-α activated endothelial cells, which in turn activates transcription of chemokine CXCL10/IP-10. This chemokine induces recruitment of various inflammatory cells such as Th1 (type-1 T helper) CD4^+^ T cells, CD8^+^ cytotoxic effector cells, natural killer, natural killer T cells, plasmocytoid dendritic cells, and some B-cell subsets to the site of inflammation [72]. Furthermore, the same group reported in a follow up study that PRMT5-mediated dimethylation of NF-κB/p65 at R174 in TNF-α and interferon-γ co-stimulated endothelial cells promotes transcription of chemokine CXCL11 [73]. Thus, understanding further the role of PRMT5-mediated methylation of HOXA9 and NF-κB/p65 may provide important mechanistic insight into how inflammation can be modulated.

## CONCLUSIONS

PRMT5 has emerged as an important biomarker for several types of cancer, and increasing evidence argues that enhanced expression of PRMT5 induces proliferative signaling, which drives cancer cell growth and survival. The oncogenic properties of PRMT5 are mediated by its ability to methylate both histone and non-histone proteins, which trigger changes necessary to reprogram cell growth from normal to malignant and ultimately metastatic. However, it is important to consider the fact that PRMT5 is also essential for normal development and differentiation. So, while it is justified to develop very potent PRMT5-specific inhibitors to treat various types of cancer, rigorous testing in cell culture and animal models should be done to rule out the adverse effects PRMT5 inhibition might have on other vital processes such as embryogenesis, spermatogenesis, neurogenesis, muscle regeneration, adult hematopoiesis and immune response. The ability of PRMT5 to methylate symmetrically specific and ubiquitous receptors, signaling molecules, transcription factors and histones, endows it with the capacity to transform the growth characteristics of almost any cell. Hence, as new information about the role played by PRMT5 in regulating cellular growth and proliferation becomes available, studies aimed at evaluating the efficacy of existing inhibitors, which can modulate other growth regulatory pathways, in combination with PRMT5 inhibitors should be conducted in cancer cells with elevated PRMT5 activity. While drug development efforts are ongoing to tailor more potent and specific inhibitors against different members of the PRMT family, which have been implicated in disease states, it is worth noting that encouraging results have been obtained with PRMT5 inhibition *in vitro* and in preclinical murine models of various aggressive and incurable malignancies. In light of these positive results, it is going to be important to develop an experimental therapeutic program that can facilitate translational research, which explores PRMT5 inhibition both alone and in combination with other drugs known to inhibit cancer cell growth.

## Abbreviations

PRMT5: Protein arginine methyltransfease 5
SAM: S-adenosylmethionine
H3R8: histone H3 arginine 8
H4R3: histone H4 arginine 3
ST7: Suppressor of Tumorigenicity 7
RB: Retinoblastoma
PTPROt: Protein Tyrosine Phosphatase Receptor-type O
PPARγ2: Peroxisome Proliferative-Activated Receptor γ2
Wnt: Wingless/Integration 1
Dlk-1: Delta Like Non-Canonical Notch Ligand 1
FGFR-3: Fibroblast-derived Growth Factor Receptor-3
eIF4E: Eukaryotic elongation Initiation Factor-4E
RPS10: ribosomal protein S10
RAF: Rapidly Accelerated Fibrosarcoma
miR: microRNA
HDAC3: Histone Deacetylase 3
CDK4: Cylcin Dependent Kinase 4
PRC2: Polycomb Repressor Complex 2
EZH2: Enhancer of Zeste Homolog 2
SUZ12: Suppressor of Zeste 12
EED: Embryonic Ectoderm Development
CASP10: Caspase 10
DAP1: Death Associated Protein 1
HOXA5: Homeobox A5
HRK: Harakiri
KLF4: Kruppel-like factor 4
MCF-7: Michigan Cancer Foundation-7
SOX9: SRY (sec determining region Y)-box 9
BMI1: Bcell-specific Moloney murine leukemia virus integration site 1
PI3K: phosphatidylinositol 3-kinase
ASK1: Apoptosis Signal-regulating Kinase 1
PDCD4: Programmed Cell Death 4
SREBP: Sterol Regulatory Element-Binding Protein
GSK3β: Glycogen Synthase Kinase 3 beta
HCC: Hepatocellular Carcinoma
ccRCC: clear cell Renal Cell Carcinoma
RNF20: Ring Finger protein 20
CRTC2: CREB Regulated Transcriptional Coactivator 2
MTAP: 5-methylthioadenosine phosphorylase
MTA: Methylthioadenosine
MEP50/WDR77: Methylosome Protein 50
RIOK1: RIO kinase
STAT3: Signal Transducer And Activator of Transcription 3
FGF5: Fibroblast Growth Factor 5
LHX1: LIM Homeobox 1
FOX2: Forkhead Box 2
OCT4: Octamer binding transcription factor 4
REX1: Reduced expression protein 1
PRDM: Positive Regulatory Domain
SC1: Schwann Cell Factor 1
BUB1b: Budding uninhibited by benzimidazoles 1
Id2: Inhibitor of DNA binding 2
STRA8: Stimulated by Reinoic Acid 8
SCP3: Synaptonemal Complex Protein 3
SEP1: Septin 1
REC8: Meiotic recombination protein REC8 homolog
HSC: Hematopoietic Stem Cells.
